# Metagenomic approaches reveal differences in genetic diversity and relative abundance of nitrifying bacteria and archaea in contrasting soils

**DOI:** 10.1038/s41598-021-95100-9

**Published:** 2021-08-05

**Authors:** Ian M. Clark, David J. Hughes, Qingling Fu, Maïder Abadie, Penny R. Hirsch

**Affiliations:** 1 Sustainable Agriculture Sciences Department, Rothamsted Research, Harpenden, AL5 2JQ Hertfordshire UK; 2 Computational and Analytical Sciences, Rothamsted Research, Harpenden, AL5 2JQ Hertfordshire UK; 3 College of Resources and Environment, Huazhong Agricultural University, Wuhan, 430070 Hubei Province People’s Republic of China

**Keywords:** Microbiology, Ecology

## Abstract

The abundance and phylogenetic diversity of functional genes involved in nitrification were assessed in Rothamsted field plots under contrasting management regimes—permanent bare fallow, grassland, and arable (wheat) cultivation maintained for more than 50 years. Metagenome and metatranscriptome analysis indicated nitrite oxidizing bacteria (NOB) were more abundant than ammonia oxidizing archaea (AOA) and bacteria (AOB) in all soils. The most abundant AOA and AOB in the metagenomes were, respectively, *Nitrososphaera* and *Ca.* Nitrososcosmicus (family Nitrososphaeraceae) and *Nitrosospira* and *Nitrosomonas* (family Nitrosomonadaceae). The most abundant NOB were Nitrospira including the comammox species *Nitrospira inopinata, Ca.* N. nitrificans and *Ca*. N. nitrosa. Anammox bacteria were also detected*.* Nitrospira and the AOA Nitrososphaeraceae showed most transcriptional activity in arable soil. Similar numbers of sequences were assigned to the *amoA* genes of AOA and AOB, highest in the arable soil metagenome and metatranscriptome; AOB *amoA* reads included those from comammox Nitrospira clades A and B, in addition to Nitrosomonadaceae. Nitrification potential assessed in soil from the experimental sites (microcosms amended or not with DCD at concentrations inhibitory to AOB but not AOA), was highest in arable samples and lower in all assays containing DCD, indicating AOB were responsible for oxidizing ammonium fertilizer added to these soils.

## Introduction

Soil microorganisms are essential for maintaining supplies of bioavailable N for plants, whether in natural ecosystems or agricultural systems^1^. A key step in the nitrogen cycle is nitrification, the oxidation of ammonia derived from breakdown of organic matter, animal wastes or man-made fertilizer to nitrate via hydroxylamine and nitrite^2^. The positively-charged ammonium cation (the predominant form of ammonia in soil) is relatively immobile, associating with negatively charged surfaces. The more mobile nitrate anion is more likely to reach roots but is liable to be lost by leaching from soil and is also the substrate for denitrification, an anaerobic process which results in losses of the greenhouse gas nitrous oxide^3^. Bacteria and archaea containing the enzyme ammonia monooxygenase (AMO) catalyse production of hydroxylamine from ammonia, usually considered to be the rate-limiting step in nitrification^2,4^. A variety of compounds such as dicyandiamide (DCD) inhibit AMO in bacteria and are applied to soil to slow conversion of fertilizer-N, limiting losses and improving the efficiency of agricultural production^5^. The relative susceptibility of AOA and AOB to commercial nitrification inhibitors in soil is not fully known but in lab culture, a representative of the most abundant AOA soil genus was unaffected by DCD at a dose five-fold higher than that needed to completely inhibit the most abundant AOB genus^6^.

Ammonia oxidizing bacteria (AOB) belong to the betaproteobacterial family Nitrosomonadaceae and the gammaproteobacterial genera *Candidatus* Nitrosoglobulus terrae and *Nitrosococcus*^7,8^. They are obligate chemoautotrophs that use type I RuBisCO to fix CO_2_ via the Calvin cycle and grow very slowly in laboratory culture as a consequence of the low energy yield from oxidation of ammonia^2,7^. The Nitrosomonadaceae are the dominant AOB in soil and two genera are currently recognised: *Nitrosospira* (which now includes *Nitrosovibrio* and *Nitrosolobus,* previously classified as separate genera) and *Nitrosomonas*^7^.

The ammonia oxidizing archaea (AOA) are considered to be oligotrophic chemoautotrophs. Currently, five major AOA lineages are recognised: Nitrososphaerales (NS); Nitrosopumilales (NP); Candidatus Nitrosocaldales (NC); Candidatus Nitrosotaleales (NT); and a separate deeply-branching group related to NP and NT^9^. In soil, the dominant lineage is NS which contains the genus *Nitrososphaera* and a related *Candidatus* genus, Nitrosocosmicus but NP and NT have also been detected^10^. *Nitrososphaera* isolates are difficult to grow in the lab but culture experiments have shown that some isolates are not obligate chemoautotrophs^11–13^. Complete ammonia oxidation to nitrate (comammox) has been demonstrated in bacteria belonging to sublineage II of the monophyletic clade Nitrospira, previously thought to comprise only nitrite oxidizers^14,15^. The enzyme AMO is distinct in AOB, AOA and comammox bacteria, the genes readily identified using metagenomics, qPCR and amplicon sequencing, providing evidence that AOA are often more abundant than AOB and respond differently to land management^16–19^. The comammox *amoA* genes fall into two clades, A and B, the former found in *Nitrospira* that include the named comammox species *N. inopinata, Ca.* N. nitrificans and *Ca*. N. nitrosa; the latter in a range of environmental *Nitrosospira* metagenomes^20,21^. Gene exchange between the betaproteobacterial AOB and Nitrospira may explain similarities in *amoA* sequences from the two phyla^22^. Other groups that oxidize ammonia using different mechanisms are the anaerobic ammonia oxidizing (anammox) Planctomycetes in marine environments and wastewater^23^ and certain heterotrophic bacteria and fungi in acid soils^24^.

The AMO in some archaea have lower ammonia saturation and inhibition constants than those in bacteria, indicating that they would benefit less from the relatively high levels of ammonia in agricultural soils^11^, although the cultured soil archaeon *Nitrososphaera viennensis* grows at levels similar to those optimal for soil AOB^25^. The mechanism by which hydroxylamine is oxidized to nitrite is also different, with no evidence in AOA for the hydroxylamine oxidoreductase (HAO) present in AOB^26–28^. The comammox *N. inopinata* also has a high ammonia affinity and low maximum ammonia oxidation rate^27^. However, increasing ammonia in soil is reported to increase the abundance of clade A comammox, AOA and AOB in agricultural soil^29^ and both comammox and AOB in forest soils^30^, indicating soil and site differences in nitrifier communities and need for further investigation into comammox ecology.

Chemoautotrophic bacteria that oxidize nitrite to nitrate (NOB), generating energy for aerobic growth, belong to the phyla Proteobacteria, Chloroflexi, Nitrospina, and Nitrospira (which includes the commamox group)^31^. The nitrite oxidoreductases (NXR), molybdopterin-containing members of the large DMSO reductase family^32^, are located in the cytoplasm of proteobacterial NOB and the periplasm of the other phyla. The distinction between nitrate reductase encoded by *narG* and the cytoplasmic NXR encoded by *nxrA* is defined by their cellular environments; however, the genes also fall into distinct groups in a phylogenetic analysis of the DMSO reductase superfamily^32^. Periplasmic NXR, as found in the Nitrospira, is estimated to be more energy-efficient than the cytoplasmic version, enabling growth at lower nitrite concentrations^33^. Whereas the proteobacterial *Nitrobacter* fix CO_2_ via the Calvin cycle, at least some Nitrospira can use organic C^34^.

The inherent difficulty in growing these slow-growing microorganisms in lab culture has previously impeded research. A better understanding of the various nitrifier groups in agricultural soil, essential for effective management of the N cycle, is now being developed using metagenomics and metatranscriptomics. On Rothamsted farm, we have shown that AOB numbers increase in response to added N fertilizer^35^ and that AOA were more abundant in tilled soils than undisturbed areas^18^. In arable soil receiving regular N fertilizer applications, the number of *amoA* copies from AOA and AOB was shown to be similar, at 5 × 10^6^ copies g^−1^ dry soil, using qPCR^36^. However, qPCR is limited by the specificity of primers, may not capture all groups that are present, may overestimate abundance of taxa with multiple 16S rRNA genes, and can be skewed by primer bias. Estimates of microbial community diversity based on 16S rRNA gene amplified sequence variants (ASVs) are used widely but are misleading when functional genes of interest are not always present in taxa defined by 16S genes ^37^. In contrast, sequencing the soil metagenome and metatranscriptome provides a representative sample of the genomes present and the genes that are being expressed although there may be an initial bias in nucleic acid extraction efficiency from different organisms, a problem for all molecular methods and subsequent analyses^37^. Current next-generation sequencing methods present different challenges due to the short reads and large data sets but the ever-expanding database of microbial genome sequences enables identification of many different taxa^37^. Here, we investigate metagenomic and metatranscriptomic datasets from Rothamsted soils with contrasting long-term management including N-fertilizer treatments, together with assays for nitrification potential, to compare the abundance and activity of bacteria and archaea involved in different nitrification steps. This includes both the overall abundance of genomic sequences belonging to taxa capable of nitrification, and sequences identified as *amoA* belonging to these groups.

## Results

In the three years prior to the experiment (2008–2010), mean DNA yields from samples taken from the field for bare fallow, arable and grassland were 21, 95 and 298 µg g^-1^ dry soil, respectively with no significant differences between replicate plots within each treatment and highly significant differences between treatments (*F*_2,18_ = 646, *P* < 0.001, all means significantly different at *α* = 0.05) and no interaction between treatments and years, indicating that the effects of treatments and time were independent. The corresponding 16S rRNA gene copy numbers were 3.4 × 10^8^, 14.1 × 10^8^ and 33.3 × 10^8^ copies g^−1^ dry soil, equivalent to approximately 85 × 10^6^, 353 × 10^6^ and 831 × 10^6^ prokaryotic cells g^-1^ dry soil assuming an average of four 16S rRNA gene copies per cell^38^, highly significantly different between treatments (*F*_2,18_ = 235, *P* < 0.001; all means significantly different at *α* = 0.05) with no effect of the sampling year and no treatment by year interaction.

In 2011 the mean DNA yields for bare fallow, arable and grassland were 22, 104 and 275 µg g^-1^ dry soil, respectively, all significantly different from each other but not from the same plots for the previous three years with no interaction between treatment and years, which justified using the three-year mean estimate of prokaryotic cells.

Metagenomic DNA sequencing generating a 149 nt average read length provided ~ 42 Gb from bare fallow and arable plots and 74 Gb from the grassland soil, corresponding to 3 × 10^8^ and 5 × 10^8^ sequences, respectively. The RNA extracted at the same time (average read length 87 nt) gave 1 × 10^8^, 2 × 10^8^ and 3 × 10^8^ sequences corresponding to 5.4, 2.9 and 8.6 Gb from bare fallow, arable and grassland soil, respectively. MEGAN6 identified over 5500 prokaryotic taxa in each of the sequenced samples (bare fallow—5617; arable—5563; grass—5516); not significantly different between treatments although the grassland sequences required the smallest mass of soil and bare fallow the largest to achieve the same amount of DNA for sequencing.

**Figure 1:**
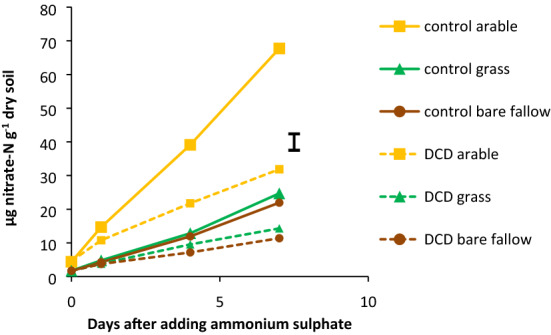
Production of nitrate in response to 100 mg ammonium-N g^−1^ dry soil added to microcosms incubated for 7 d with no inhibitors (solid lines) or 200 µM dicyandiamide (DCD) (dashed lines); standard error of difference of means (*P* < 0.001) shown as bar.

Functional nitrification assays showed that after 7 days, 70% of the added ammonium-N had been converted to nitrate in the arable soil compared to only 20% in bare fallow and grassland soils. The daily rate of nitrate production was approximately threefold higher in arable soil; DCD at concentrations that specifically inhibit bacterial AMO, halved the rate of nitrate production in all soils (Fig. 1).

**Figure 2:**
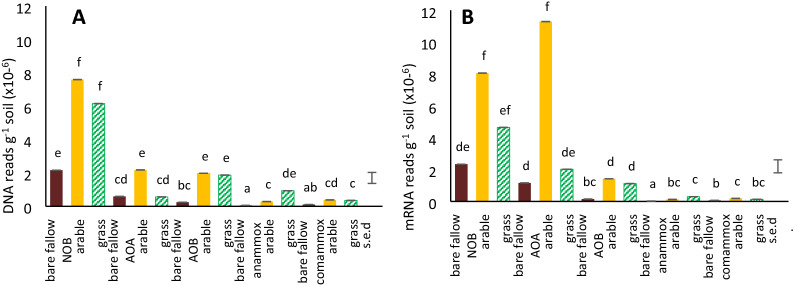
Mean relative abundance: (**A**)—DNA and (**B**)—mRNA reads g^-1^ dry soil assigned to nitrifying guilds in each soil treatment. Standard errors of differences of means (s.e.d.) shown for all groups; different letters indicate significantly different means.

Metagenomes and metatranscriptomes were interrogated using DIAMOND/MEGAN6 for all sequences belonging to known guilds AOA, AOB, NOB, comammox and anammox. NOB sequences were significantly most abundant overall in the metagenome with similar numbers for AOA and AOB, and significantly fewer for comammox and anammox bacteria (*F*_4,30_ = 139, *P* < 0.001); NOB and AOA sequences had similar abundance in the transcriptome, significantly more than AOB which in turn was significantly more abundant than comammox and anammox (*F*_4,30_ = 230, *P* < 0.001; (Fig. 2A, B). Overall, DNA sequences assigned to nitrifying groups were significantly more abundant in the arable and grassland soils than in bare fallow (*F*_2,30_ = 119, *P* < 0.001; Fig. 2A) whereas there were most mRNA sequences in the arable soil with fewer in grass and fewest in the bare fallow soil (*F*_2,30_ = 111, *P* < 0.001; Fig. 2B). When the results were expressed as ‰, NOB and AOA DNA formed a significantly greater proportion of the total prokaryotic sequences in arable and bare fallow compared to the grassland soil (*F*_2,30_ = 139, *P* < 0.001; Supplementary Fig. 1A), and mRNA ‰ was significantly highest in arable and lowest in bare fallow soil (*F*_2,30_ = 230, *P* < 0.001; Supplementary Fig. 1B).

**Figure 3:**
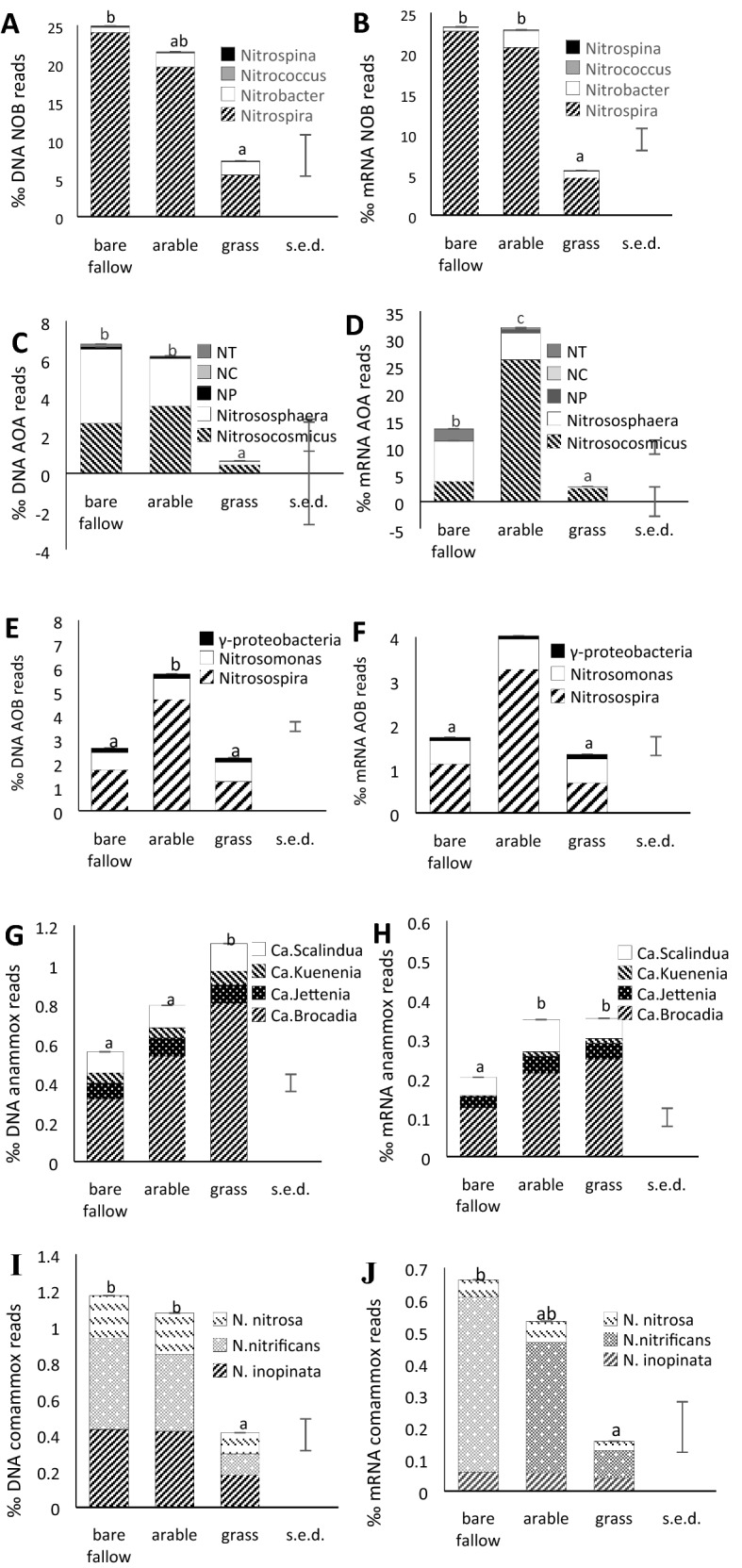
Mean proportion of DNA reads (left) and mRNA (right) reads assigned to the main nitrifying guilds shown in Fig. 2, expressed as per 1000 of total. The proportions formed by the component groups of each guild are shown (ANOVA statistics of component groups are shown in supplementary tables 1 and 2). Overall standard errors of differences of means (S.E.D.) are shown for each guild; different letters indicate significantly different means for each guild in each treatment. (**A**, **B**) – NOB; (**B**, **C**)—AOA; (**E**, **F**)—AOB; (**G**, **H**)—anammox; (**I**, **J**)—comammox.

Comparison of DNA and mRNA reads of known nitrifier guilds identified using DIAMOND/MEGAN6 revealed that the NOB were dominated by *Nitrospira,* with *Nitrobacter* comprising 2–20% and the other NOB groups less than 2% (Fig. 3A, B). For AOA, the NS groups *Ca.* Nitrosocosmicus and *Nitrososphaera* were most numerous*,* with *Ca.* Nitrosocosmicus more dominant in the mRNA and fewer than 5% of sequences assigned to other groups (Fig. 3C, D). The most abundant AOB in soil metagenomes and metatranscriptomes was *Nitrosospira* (50—80%) with fewer assigned to *Nitrosomonas* (13–41%) and < 10% identified as gammaproteobacteria (Fig. 3E, F).

Anammox bacterial DNA and mRNA was dominated by *Ca.* Brocadia but all known genera were detected (Fig. 3G, H). However, the overall proportion of comammox and anammox bacteria was low compared to the AOA, AOB and NOB (Fig. 2A, B). Of NOB identified as comammox, *N. inopinata* and *Ca.* N. nitrificans were present with similar abundance in DNA reads with *Ca.* N. nitrosa present at 20–28% but in mRNA, *Ca.* N. nitrificans was most abundant at 55–83% of the total (Fig. 3I,J). The named comammox species were present at around 5% of total Nitrospira DNA and 3% of mRNA sequences, but they were closer in abundance to the AOB in bare fallow soils (~ 40% of DNA and mRNA sequences, 20% DNA 10% RNA in arable and grassland). A full list of mean DNA and mRNA values for each group within each guild, and the guild totals, expressed both as reads g^−1^ dw soil and as ‰ total prokaryotic DNA or mRNA sequences in each sample, together with ANOVA results and Tukey’s post-hoc test for significantly different means, is given in Supplementary Tables 1–4.

**Figure 4:**
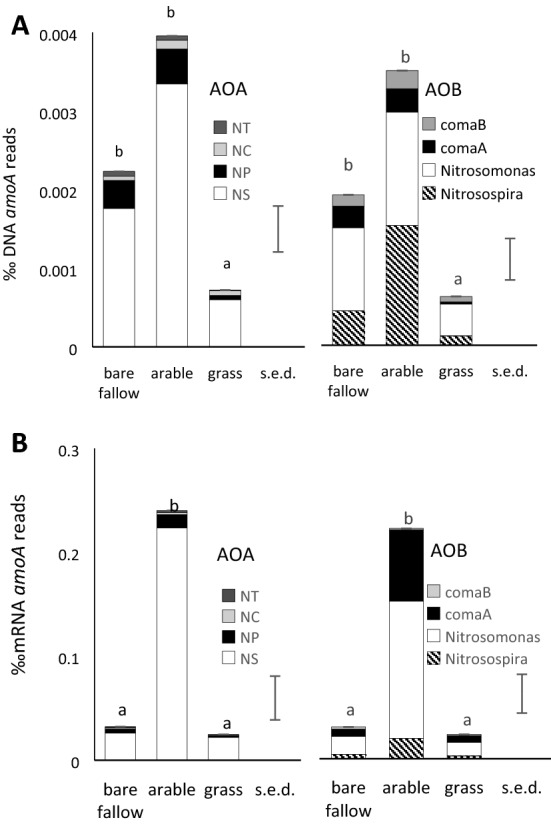
Mean proportion of (**A**)—DNA reads and (**B**)—mRNA *amoA* reads assigned to the AOA and AOB (including comammox). The proportion of the total formed by each sub-group identified is shown (the NS AOA could not be separated into *Nitrososphaera* and *Ca.* Nitrosocosmicus). ANOVA statistics of the component groups are shown in supplementary table 5.

Further analyses identified individual sequence reads with homology to *amoA* genes from AOA, AOB and comammox clades A and B in the metagenomes and metatranscriptomes. The proportion of reads assigned to the component groups of each guild, expressed as ‰ total prokaryotic sequences, are shown in Fig. 4A, B and Supplementary Table 5. Results expressed as reads g^-1^ dw soil are shown in Supplementary Fig. 2A, B and Supplementary Table 6. The number of genomic sequences assigned to AOA and AOB *amoA* was < 0.1% of the overall total number of DNA sequences assigned to the genome of each group. However, number of mRNA reads was higher, with AOB *amoA* reads at 6% of the total for AOB in arable soil, 2% in bare fallow and 1.5% in grassland soil; for AOA *amoA* there were < 1% total AOA mRNA reads in all soils. AOA *amoA* could not be divided into sequences belonging to either *Nitrososphaera* or *Ca.* Nitrosocosmicus, but the NS group was the most abundant around 80% of DNA and 90% of mRNA reads, with NP comprising 8 – 16% of DNA and 5–13% mRNA reads. AOB *amoA* DNA sequences included *Nitrosospira* and *Nitrosomonas* comprising 60% of DNA reads with comammox clades A and B contributing 12–16%. The Nitrosomonadaceae also contributed 60% of mRNA reads with commamox clade A contributing 20–27% and gammaproteobacterial 10–14%. No reads were detected from the gammaproteobacterial AOB *Ca.* Nitrosoglobulus terrae and *Nitrosococcus.*

## Discussion

Our previous finding, based on 16S rRNA amplicon sequencing, indicated that AOA constitute a higher proportion of the microbial community in tilled soils than undisturbed areas^18^ and that the Nitrospira were more abundant than the AOA, with highest numbers in the arable plots^36^. The AOA and AOB were more abundant in the Highfield arable soils compared to grassland and bare fallow, using qPCR, where ~ 5 × 10^6^ copies of AOA-*amoA* and AOB-*amoA* g^-1^ dry arable soil with fewer in the other plots^36^. In our current study, the relative abundance of AOA and AOB reads in the metagenomic and metatranscriptome gave similar results, with an estimated of ~ 3 × 10^6^ AOA and AOB in arable soil. It also indicates that the AOA are more transcriptionally active than the AOB, possibly because AOA remain active when N levels in soil are low whereas AOB are inactive in October, when soil was sampled prior to fertilizer addition. The values were 0.09, 0.15 and 0.34%N for bare fallow, arable and grassland soils, respectively (corresponding soil organic C was 0.85, 1.64 and 3.09%C)^36^. The ability of AOA to survive heterotrophically, as well as their high ammonia affinity enabling utilization of very low concentrations, may contribute to this. However, the relative abundance of *amoA* DNA and mRNA reads from AOA and AOB was similar, with mRNA comprising a much larger proportion of the metatranscriptome (2‰) than DNA reads of the metagenome (0.003‰), indicating the importance of AMO for these nitrifiers. The AOB-*amoA* mRNA included a relatively high proportion of comammox (40—50%), broadly consistent with the relative abundance of the total number of reads assigned to the Nitrosomonadaceae and clade A comammox Nitrospira overall (10–60%). Results may be influenced by the assignment of reads from Nitrosomonadaceae to comammox Nitrospira, or *vice-versa*, due to similarities between their *amoA* genes^22^, and potential biases in databases used for the assignment. However, they support recent reports based on different methods (qPCR, DNA-stable isotope probing) showing that comammox are present and active in relatively high numbers in soil^29,30^. Our study also found evidence for *amoA* DNA sequences (with low levels of corresponding mRNA) belonging to clade B comammox Nitrospira, not previously reported in soil.

The functional assay for nitrification potential, where ammonium was added to soil at a relatively high concentration equivalent to 250 kg N ha^-1^, showed much higher enzyme activity attributed to AOB (i.e. inhibited by DCD) in the arable soil. Nitrification activity in grassland and bare fallow soil was similar although AOB were more abundant in grassland. The concentration of DCD was calculated to be inhibitory to the AOB *Nitrosospira amoA* but not to affect the AOA *Nitrososphaera amoA*, based on a previous study of the Highfield soils^39^. Nitrification commences rapidly after substrate addition and this assay measures AMO already present in cells, rather than *de-novo* transcription of *amoA* and synthesis of new enzyme, or cell division^40^. Experiments with longer incubation periods and lower DCD concentrations reported that growth of clade A comammox Nitrospira was inhibited in grassland but not arable soil; AOA and AOB were inhibited in both soils^41^.

Our study shows that AOA are both more numerous and active than AOB, especially in N-fertilized arable soils compared to bare fallow with no N inputs and grassland containing large populations of competitive hetrotrophic bacteria. The AOB are likely to be responsible for most of the rapid nitrification observed when N fertilizer is added to soil but appear to be less active when N-availability is low; AOA in contrast remain active at low soil N concentrations and may provide a background level of nitrification. It is unclear if the comammox AOB are contributing to nitrification although they are present and active at around 50% of the number of Nitrosomonadaceae in the arable soil. Since the AMO of comammox AOB is similar to that of other AOB^22^, it is likely that both are inhibited by DCD. Other reports implicate clade A comammox AOB in nitrification in both agricultural and forest soil^30,41^.

The Nitrosomonadaceae are obligate autotrophs and obtain C only from CO_2_ fixation via the Calvin cycle; the AOA and comammox Nitrospira use different and more efficient C fixation pathways and at least some can utilize simple organic substrates^29,42^. Therefore, at least in Rothamsted soils, the AOA and comammox AOB have a survival advantage in soil conditions where organic C is available and the paucity of N limits the Nitrosomonadaceae. However, when N fertilizer is applied, they may be less able to take advantage of the increased ammonia concentration than the Nitrosomonadaceae, which increase in numbers.

The NOB are present and active in relatively high numbers compared to the AOA and AOB, which may explain why nitrite oxidation is rapid and rarely limiting in most soil. Nitrospira are the most abundant group, with *Nitrobacter,* previously considered to be the archetypal nitrifier, at < 20%. This suggests that further investigation of the role of Nitrospira in soil is important. In contrast to the cytoplasmic NXR of *Nitrobacter*, the periplasmic Nitrospira NXR maintains oxidation activity at very low nitrite concentrations^33^. The Nitrospira fix CO_2_ more efficiently than *Nitrobacter* (which, like the proteobacterial AOB*,* use the Calvin cycle) and, as mentioned above, can utilize some organic C^42^ potentially providing an alternative energy source^34^. This would enable Nitrospira to compete in low nutrient soils and benefit from nitrite generated by AOA also active in those conditions. Meanwhile, it is likely that the proteobacterial nitrifiers Nitrosomonadaceae and *Nitrobacter* take advantage of the relatively high ammonia levels after N fertilizer is applied to arable soil with a growth boost but then die down as plants remove the N and become less abundant than the AOA and Nitrospira at the end of the season, as in October when soil was sampled. Our previous work had shown such episodic increases in AOB in response to fertilizer applications^35^. Thus, AOA and AOB (including comammox), Nitrospira and *Nitrobacter* may not compete directly for resources in soil, but instead occupy complementary niches. Confirmation of this needs further studies in a range of geographical locations and land uses, and different soils, management systems and climates may support alternative communities and exhibit contrasting nitrifier ecology.

The anammox bacteria were not thought to play a role in aerobic environments such as soil, and their numbers are relatively low, but they appear to be present and active and it is possible that they inhabit anaerobic, saturated soil pores and have an as-yet undetermined role in the terrestrial N cycle. The study also revealed groups previously reported in marine environments, including. the NP AOA Nitrosopumulis and the NOB Nitrospina, were present at low abundance in terrestrial systems, supporting reports that used PhyloChip microarrays to identify taxa^43^.

The analysis of soil metagenomes and metatranscriptomes offers a new approach to understanding soil microbial ecology and this study indicates that the results are consistent with those obtained using other methods to target specific taxa such as microarrays, ASVs and qPCR. Extracting and sequencing all soil DNA and mRNA prior to bioinformatic analysis avoids some of the biases inherent in other methods and adds to a more comprehensive understanding of the functioning of the complex soil microbiome.

## Methods

### Soil sampling

The Highfield Ley-Arable experiment at Rothamsted Research contrasts different long-term land management: permanent grassland; continuous arable (wheat) that receives regular fertilizer applications; and bare fallow, where all plant growth has been removed by regular tillage. Located at 00:21:48 W, 51:48:18 N, the soil is a silty loam over clay and is classified as a Chromic Luvisol according to FAO criteria. After > 50 years, the plots have developed distinct microbial communities and divergent soil organic C and structure^44^. Soil was collected from the Highfield permanent plots in October 2011 to 10 cm depth using a 3 cm diameter corer; the top 2 cm containing root mats and other plant detritus was discarded. Ten cores per plot were pooled, thoroughly mixed whilst sieving through 2 mm mesh and placed in liquid N_2_ within 5 min of collection; then samples were frozen at -80 °C prior to nucleic acid extraction; this provided three true replicate soil samples per plot treatment. All implements were cleaned with 70% ethanol between sampling/sieving soil from each plot.

### Nucleic acid extraction and analysis

To ensure that extracted DNA and RNA came from the same soil sample, community DNA and RNA was extracted from a minimum of 2 g soil using the MoBio RNA PowerSoil® Total RNA isolation kit followed by the RNA PowerSoil®DNA Elution Accessory kit, with three replicates for each soil treatment. All RNA samples were DNAase treated with Ambion Turbo DNA-free™_._ When necessary, extracts were pooled to provide sufficient material for sequencing. For the three years prior to this study, soil 16S rRNA gene copy numbers were estimated by qPCR with universal primers to allow normalisation of results^45^.

Full metagenomic sequencing of > 10 µg DNA from each replicate soil treatment was provided by Illumina®, Cambridge, UK using a HiSeq 2000, generating 150 bp paired end reads. RNA was subjected to ribodepletion and sequenced by The Genome Analysis Centre (TGAC), Norwich, UK using a HiSeq 2000, generating 100 bp paired end reads. Sequences were quality checked using the FASTX-Toolkit (version 0.0.13.2, http://hannonlab.cshl.edu/fastx_toolkit/index.html) with a quality threshold of 25, minimum length 100 bp for DNA and 80 bp for RNA. Reads were assigned to taxa using DIAMOND^46^ and analysed in MEGAN6^47^; Megan assigned KO (KEGG Orthology) molecular functional identifiers were used to extract individual sequences for each KEGG function (k04561: norB, k10944 : amoA). Alignment and phylogenic assignation of retrieved sequences from metagenomic libaries was performed using MAFFT v7.450^48^ translation alignment and Geneious v10.2.3 Tree Builder (Jukes-Cantor distance model, Neighbor-Joining tree build, random seed 1000 bootstraps) against archetypal reference sequences across the diveristy of the targeted gene. Sequences retrieved from the soil metagenomes have been deposited in the ENA database (http://www.ebi.ac.uk/ena/data/) as project PRJEB46652/ERP13086.

The relative presence of each group or taxon assigned to taxon by DIAMOND/MEGAN6 was expressed as a proportion per 1000 (‰) of all prokaryotic reads in the relevant sample; the relative abundance of taxa in soil was estimated as the product of (number of prokaryotic cells g^-1^ dry soil) x (proportion of all reads assigned to taxon by DIAMOND/MEGAN6 as a proportion of prokaryotic reads). Taxa assumed to represent nitrifying groups are indicated in the introduction.

### Potential nitrification activity

Soil was collected from each plot in October 2014, mixed and sieved as described above then 25 g aliquots were placed in Ziploc bags. These were stored in the dark at 4 °C for 7 days and 20 °C for a further 7 days. Meanwhile samples were taken to estimate field water holding capacity and dry weight. A solution was added at levels estimated to provide 100 mg NH_4_^+^-N g^-1^ dry soil with water to standardise moisture at 60% of field capacity; for controls, water alone was added. The nitrification inhibitor DCD was added to half of the bags to provide 16.8 μg mL^-1^ in the pore water (≡200 μM). Bags were incubated in the dark at 20 °C and 5 g samples taken at the start, after 1, 4 and 7 days and frozen at -20 °C. Soils were extracted in 2 M KCl (5 mL g^-1^ dw soil) by vigorous shaking (300 rpm) for 2 h then left to stand for 45 min before filtering through Whatman no 1 paper. Nitrate and ammonium in the filtrate were analysed simultaneously using a Skalar SAN^PLUS^ System continuous flow analyser; nitrite was measured in a separate run. There were three plot treatment replicates and at least two experimental replicates per treatment.

### Statistical analyses

Statistical analysis was performed using one-factor and general ANOVA in GenStat 19th Edition (VSN International Ltd., Hemel Hempstead, UK). To check that each set of measured values met the assumptions of ANOVA and were normally distributed, residuals were plotted. If they did not show normal distribution, data was log-transformed and again checked for normal distribution of residuals. Treatment comparisons with *F* statistics with *P* < 0.05 were considered significant, *P* < 0.001 highly significant. Mean*s* were compared using Tukey’s *post-hoc* method in the GenStat multiple comparison menu with 95% confidence; means are considered significantly different at α = 0.05 and where appropriate, are represented by different letters.

## Supplementary Information

Supplementary Information.
