# Supplementary material for: Metagenomic approaches reveal differences in genetic diversity and relative abundance of nitrifying bacteria and archaea in contrasting soils
Source: Sci Rep. 2021 Aug 5;11:15905. doi: 10.1038/s41598-021-95100-9 (PMC8342464; doi:10.1038/s41598-021-95100-9)
Supplement: Supplementary file 1 — Supplementary Information. [file 41598_2021_95100_MOESM1_ESM.pdf]

Metagenomic approaches reveal differences in genetic diversity and relative abundance of nitrifying bacteria and archaea in contrasting soils

Ian M Clark, David J Hughes, Qingling Fu, Maïder Abadie, Penny R Hirsch

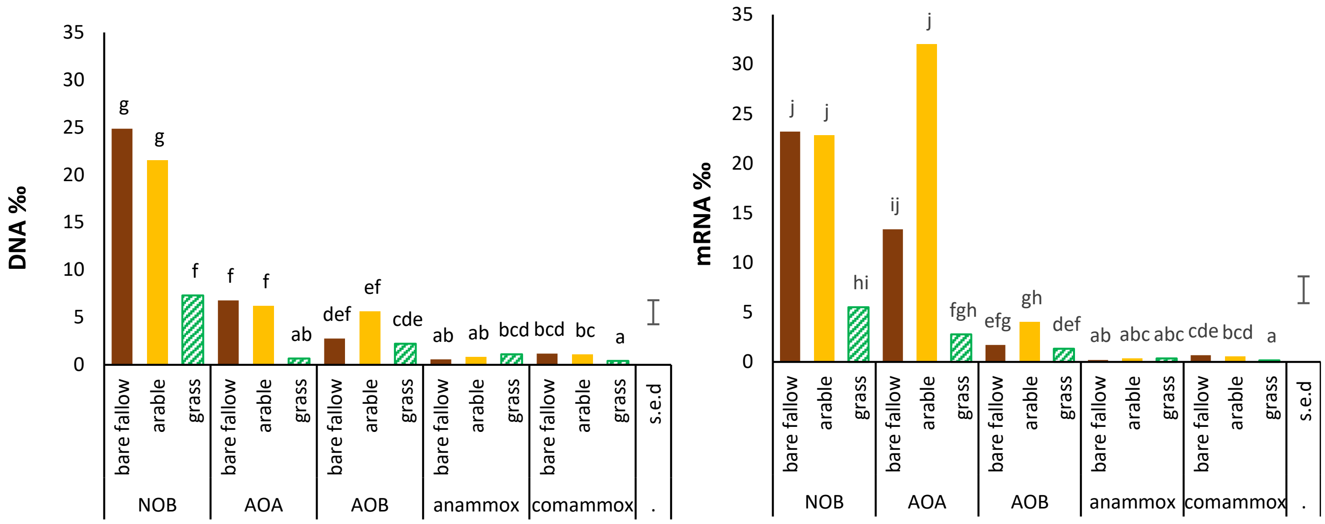

Supplementary Figure 1. Mean proportion of DNA reads (left) and mRNA (right) reads assigned to nitrifying guilds in each soil treatment, as ‰ of all prokaryotic sequences in each sample. Standard errors of differences of means (s.e.d.) shown for all guilds; different letters indicate significantly different means. ANOVA statistics for component groups are shown in supplementary tables 3, 4.

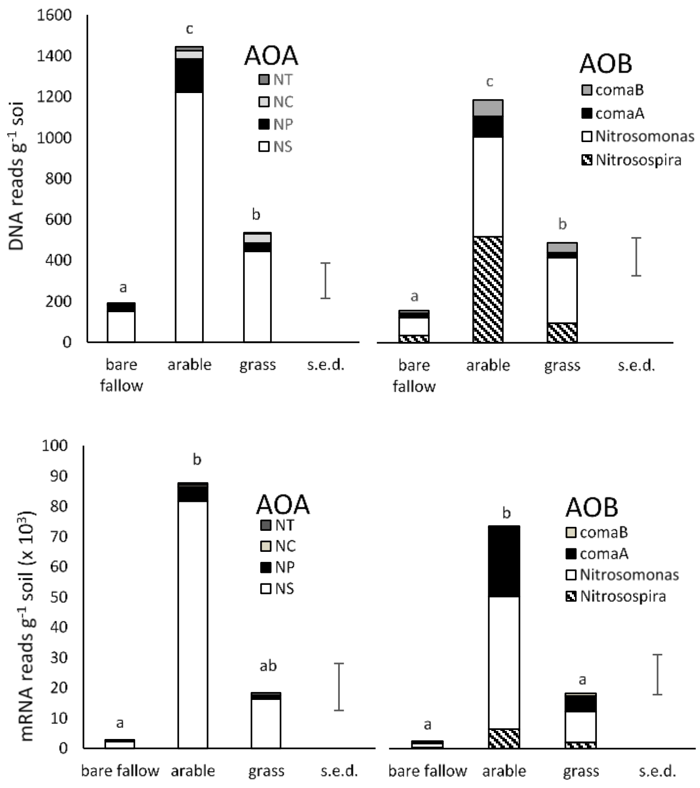

Supplementary Figure 2. Mean DNA (top) and mRNA (bottom) *amoA* reads g^{-1} dw soil assigned to the AOA and AOB including comammox in each sample. Each group is shown as a proportion of each guild in each soil treatment (the NS AOA could not be separated into *Nitrososphaera* and *Ca.* Nitrosocosmicus). ANOVA statistics for component groups are shown in supplementary table 6.

**NOB**	**DNA g^{-1}**	**Nitrospira**	***Nitrobacter***	**Nitrococcus**	**Nitrospina**	**NOB**	
**ANOVA**	$F_{2,6}$	5.95	1377.39	1752.06	539.82	8.07	
	P	0.04	<.001	<.001	<.001	0.02	
**means**	fallow	2.09E+06	6.10E+04	7.76E+03	4.52E+03	2.16E+06	
	arable	6.89E+06	6.53E+05	3.50E+04	1.99E+04	7.59E+06	
	grass	4.59E+06	1.42E+06	9.37E+04	5.25E+04	6.16E+06	
**s.e.d.**		1.39E+06	2.59E+04	1.48E+03	1.49E+03	1.40E+06	
**significantly different means**	fallow	a	a	a	a	a	
	arable	b	b	b	b	b	
	grass	ab	c	c	c	ab	
**AOA**	**DNA g^{-1}**	**Nitrosocosmicus**	***Nitrososphaera***	**NP**	**NC**	**NT**	**AOA**
**ANOVA**	$F_{2,6}$	7.76	8.92	17.9	3.54	2.27	9.56
	P	0.022	0.016	0.003	0.096	0.184	0.014
**means**	fallow	2.28E+05	3.40E+05	7.64E+03	5.52E+01	1.38E+04	5.90E+05
	arable	1.25E+06	8.87E+05	3.16E+04	3.82E+02	1.32E+04	2.18E+06
	grass	3.67E+05	1.72E+05	1.73E+04	0.00E+00	3.08E+03	5.59E+05
**s.e.d.**		2.80E+05	1.77E+05	4.03E+03	1.55E+02	5.64E+03	4.23E+05
**significantly different means**	fallow	a	a	a	a	a	a
	arable	b	b	b	a	a	b
	grass	a	a	a	a	a	a
**AOB**	**DNA g^{-1}**	***Nitrosospira***	***Nitrosomonas***	**γProteobacteria**	**AOB**		
**ANOVA**	$F_{2,6}$	127.3	466.33	920.92	148.65		
	P	<.001	<.001	<.001	<.001		
**means**	fallow	1.46E+05	7.50E+04	1.61E+04	2.37E+05		
	arable	1.65E+06	2.60E+05	6.78E+04	1.98E+06		
	grass	1.02E+06	6.71E+05	1.56E+05	1.85E+06		
**s.e.d.**		9.46E+04	2.00E+04	3.28E+03	1.12E+05		
**significantly different means**	fallow	a	a	a	a		
	arable	c	b	b	b		
	grass	b	c	c	b		
**anammox**	**DNA g^{-1}**	***Ca.*Brocadia**	***Ca.*Jettenia**	***Ca.*Kuenenia**	***Ca.*Scalindua**	**anammox**	
**ANOVA**	$F_{2,6}$	468.8	176.66	355.09	321.06	493.6	
	P	<.001	<.001	<.001	<.001	<.001	
**means**	fallow	2.78E+04	6.87E+03	4.59E+03	9.30E+03	4.85E+04	
	arable	1.89E+05	3.24E+04	1.82E+04	3.97E+04	2.80E+05	
	grass	6.78E+05	7.79E+04	6.02E+04	1.18E+05	9.34E+05	
**s.e.d.**		2.21E+04	3.83E+03	2.18E+03	4.41E+03	2.92E+04	
**significantly different means**	fallow	a	a	a	a	a	
	arable	b	b	b	b	b	
	grass	c	c	c	c	c	
**comammox**	**DNA g^{-1}**	***N. inopinata***	**N.nitrificans**	**N. nitrosa**	**comammox**		
**ANOVA**	$F_{2,6}$	10.6	13.31	35.13	13.78		
	P	0.011	0.006	<.001	0.006		
**means**	fallow	3.74E+04	4.38E+04	2.06E+04	1.02E+05		
	arable	1.48E+05	1.50E+05	8.10E+04	3.79E+05		
	grass	1.49E+05	9.90E+04	9.83E+04	3.47E+05		
**s.e.d.**		2.79E+04	2.06E+04	9.72E+03	5.78E+04		
**significantly different means**	fallow	a	a	a	a		
	arable	b	b	b	b		
	grass	b	ab	b	b		

Supplementary Table 1. Mean abundance of DNA reads g^{-1}soil of all groups in each guild (total on right) with ANOVA results. Different letters denote significantly different means (α = 0.5) according to Tukey's post-hoc method.

**NOB**	**mRNA g^{-1}**	**Nitrospira**	***Nitrobacter***	**Nitrococcus**	**Nitrospina**	**NOB**	
**ANOVA**	$F_{2,6}$	7.92	75.66	208.00	100.83	9.73	
	P	0.02	<.001	<.001	<.001	0.01	
**means**	fallow	1.97E+06	4.43E+04	5.32E+02	2.40E+03	2.02E+06	
	arable	7.29E+06	7.51E+05	9.18E+03	9.61E+03	8.06E+06	
	grass	3.87E+06	7.15E+05	3.14E+04	2.60E+04	4.65E+06	
**s.e.d.**		1.35E+06	6.47E+04	1.56E+03	1.70E+03	1.37E+06	
**significantly different means**	fallow	a	a	a	a	a	
	arable	b	b	b	b	b	
	grass	ab	b	c	c	ab	
**AOA**	**mRNA g^{-1}**	**Nitrosocosmicus**	***Nitrososphaera***	**NP**	**NC**	**NT**	**AOA**
**ANOVA**	$F_{2,6}$	31.54	59.91	17.6	nd	0.72	42.98
	P	<.001	<.001	0.003	nd	0.527	<.001
**means**	fallow	3.22E+05	6.47E+05	6.19E+03	nd	1.88E+05	1.16E+06
	arable	9.20E+06	1.75E+06	2.71E+05	nd	7.50E+04	1.13E+07
	grass	2.05E+06	2.63E+05	2.48E+04	nd	1.97E+03	2.34E+06
**s.e.d.**		1.19E+06	1.41E+05	1.30E+05	0.00E+00	1.56E+05	1.20E+06
**significantly different means**	fallow	a	a	a	nd	a	a
	arable	b	b	b	nd	a	b
	grass	a	a	a	nd	a	a
**AOB**	**mRNA g^{-1}**	***Nitrosospira***	***Nitrosomonas***	**γProteobacteria**	**AOB**		
**ANOVA**	$F_{2,6}$	38.42	40.3	499.17	106.93		
	P	<.001	<.001	<.001	<.001		
**means**	fallow	9.45E+04	4.53E+04	6.25E+03	1.46E+05		
	arable	1.15E+06	2.43E+05	2.60E+04	1.42E+06		
	grass	5.65E+05	4.57E+05	8.46E+04	1.11E+06		
**s.e.d.**		1.21E+05	4.58E+04	2.58E+03	1.53E+05		
**significantly different means**	fallow	a	a	a	a		
	arable	c	b	b	b		
	grass	b	c	c	b		
**anammox**	**mRNA g^{-1}**	***Ca.*Brocadia**	***Ca.*Jettenia**	***Ca.*Kuenenia**	***Ca.*Scalindua**	**anammox**	
**ANOVA**	$F_{2,6}$	54.14	38.56	70.51	57.62	105.51	
	P	<.001	<.001	<.001	<.001	<.001	
**means**	fallow	1.09E+04	2.35E+03	3.06E+02	4.07E+03	1.76E+04	
	arable	7.51E+04	1.51E+04	4.11E+03	2.89E+04	1.23E+05	
	grass	2.09E+05	3.33E+04	1.08E+04	4.35E+04	2.97E+05	
**s.e.d.**		1.95E+04	3.54E+03	8.92E+02	3.72E+03	1.94E+04	
**significantly different means**	fallow	a	a	a	a	a	
	arable	b	b	b	b	b	
	grass	c	c	c	c	c	
**comammox**	**mRNA g^{-1}**	***N. inopinata***	**N.nitrificans**	**N. nitrosa**	**comammox**		
**ANOVA**	$F_{2,6}$	38.69	20.58	10.58	20.51		
	P	<.001	0.002	0.011	0.002		
**means**	fallow	5.04E+03	4.78E+04	4.74E+03	5.76E+04		
	arable	1.96E+04	1.45E+05	2.25E+04	1.88E+05		
	grass	3.41E+04	7.22E+04	2.48E+04	1.31E+05		
**s.e.d.**		3.30E+03	1.58E+04	4.77E+03	2.04E+04		
**significantly different means**	fallow	a	a	a	a		
	arable	b	b	b	b		
	grass	c	a	b	b		

Supplementary Table 2. Mean abundance of mRNA reads g^{-1}soil of all groups in each guild with ANOVA results. Different letters denote significantly different means (α = 0.5) according to Tukey's post-hoc method.

**NOB**	**DNA ‰**	**Nitrospira**	***Nitrobacter***	**Nitrococcus**	**Nitrospina**	**NOB**	
**ANOVA**	$F_{2,6}$	6.49	270.77	10.38	3.41	5.98	
	P	0.032	<.001	0.011	0.102	0.037	
	fallow	24.03	0.70	0.09	0.05	24.87	
**means**	arable	19.51	1.85	0.10	0.06	21.51	
	grass	5.45	1.68	0.11	0.06	7.30	
**s.e.d.**		5.38	0.05	0.00	0.00	5.39	
**significantly different means**	fallow	b	a	a	a	b	
	arable	ab	c	ab	a	ab	
	grass	a	b	b	a	a	
**AOA**	**DNA ‰**	**Nitrosocosmicus**	***Nitrososphaera***	**NP**	**NC**	**NT**	**AOA**
**ANOVA**	$F_{2,6}$	8.28	5.46	8.97	2.29	3.37	8
	P	0.019	0.045	0.016	0.182	0.105	0.02
	fallow	2.62	3.91	0.09	0.00	0.16	6.78
**means**	arable	3.53	2.51	0.09	0.00	0.04	6.17
	grass	0.44	0.20	0.02	0.00	0.00	0.66
**s.e.d.**		0.78	0.39	0.02	0.00	0.06	1.68
**significantly different means**	fallow	ab	b	b	a	a	b
	arable	b	ab	b	a	a	b
	grass	a	a	a	a	a	a
**AOB**	**DNA ‰**	***Nitrosospira***	***Nitrosomonas***	**γProteobacteria**	**AOB**		
**ANOVA**	$F_{2,6}$	75.11	0.77	0.03	39.74		
	P	<.001	0.502	0.969	<.001		
	fallow	1.71	0.73	0.19	2.78		
**means**	arable	4.67	0.88	0.19	5.59		
	grass	1.23	0.81	0.19	2.22		
**s.e.d.**		0.30	0.12	0.02	0.41		
**significantly different means**	fallow	a	a	a	a		
	arable	b	a	a	b		
	grass	a	a	a	a		
**anammox**	**DNA ‰**	***Ca.*Brocadia**	***Ca.*Jettenia**	***Ca.*Kuenenia**	***Ca.*Scalindua**	**anammox**	
**ANOVA**	$F_{2,6}$	28.63	0.27	10.01	11.46	19.88	
	P	<.001	0.775	0.012	0.009	0.002	
	fallow	0.32	0.08	0.05	0.11	0.56	
**means**	arable	0.54	0.09	0.05	0.11	0.79	
	grass	0.80	0.09	0.07	0.14	1.11	
**s.e.d.**		0.06	0.02	0.00	0.01	0.09	
**significantly different means**	fallow	a	a	a	a	a	
	arable	b	a	a	a	a	
	grass	c	a	b	b	b	
**comammox**	**DNA ‰**	***N. inopinata***	**N.nitrificans**	**N. nitrosa**	**comammox**		
**ANOVA**	$F_{2,6}$	6.52	16.47	12.95	11.08		
	P	0.031	0.004	0.007	0.01		
	fallow	0.43	0.50	0.24	1.17		
**means**	arable	0.42	0.42	0.23	1.07		
	grass	0.18	0.12	0.12	0.41		
**s.e.d.**		0.08	0.07	0.03	0.18		
**significantly different means**	fallow	b	b	b	b		
	arable	ab	b	b	b		
	grass	a	a	a	a		

Supplementary Table 3. ANOVA results and mean proportion of DNA reads in groups as ‰ total prokaryotic sequences. Different letters denote significantly different means (α = 0.5) according to Tukey's post-hoc method.

**NOB**	**mRNA ‰**	**Nitrospira**	***Nitrobacter***	**Nitrococcus**	**Nitrospina**	**NOB**	
**ANOVA**	$F_{2,6}$	6.56	61.72	14.3	0.2	6.94	
	P	0.031	<.001	0.005	0.826	0.028	
	fallow	22.68	0.51	0.01	0.03	23.23	
**means**	arable	20.66	2.13	0.03	0.03	22.84	
	grass	4.60	0.85	0.04	0.03	5.51	
**s.e.d.**		5.47	0.15	0.01	0.01	5.43	
**significantly**	fallow	b	a	a	a	b	
**different**	arable	ab	b	b	a	b	
**means**	grass	a	a	b	a	a	
**AOA**	**mRNA ‰**	**Nitrosocosmicus**	***Nitrososphaera***	**NP**	**NC**	**NT**	**AOA**
**ANOVA**	$F_{2,6}$	63.49	56.38	13.27	nd	0.95	65.05
	P	<.001	<.001	0.006	nd	0.437	<.001
	fallow	3.70	7.44	0.07	nd	2.16	13.37
**means**	arable	26.06	4.95	0.77	nd	0.21	32.00
	grass	2.43	0.31	0.03	nd	0.00	2.78
**s.e.d.**		2.36	0.68	0.37	nd	1.72	2.59
**significantly**	fallow	a	c	a	nd	a	b
**different**	arable	b	b	b	nd	a	c
**means**	grass	a	a	a	nd	a	a
**AOB**	**mRNA ‰**	***Nitrosospira***	***Nitrosomonas***	**γProteobacteria**	**AOB**		
**ANOVA**	$F_{2,6}$	32.05	1.4	9.67	23.26		
	P	<.001	0.316	0.013	0.001		
	fallow	1.11	0.53	0.07	1.71		
**means**	arable	3.27	0.69	0.07	4.03		
	grass	0.68	0.55	0.10	1.33		
**s.e.d.**		0.35	0.10	0.01	0.43		
**significantly**	fallow	a	a	a	a		
**different**	arable	b	a	a	b		
**means**	grass	a	a	b	a		
**anammox**	**mRNA ‰**	***Ca.*Brocadia**	***Ca.*Jettenia**	***Ca.*Kuenenia**	***Ca.*Scalindua**	**anammox**	
**ANOVA**	$F_{2,6}$	6.09	3.76	15.64	5.51	6.99	
	P	0.036	0.087	0.004	0.044	0.027	
	fallow	0.12	0.03	0.00	0.05	0.2021	
**means**	arable	0.21	0.04	0.01	0.08	0.349	
	grass	0.25	0.04	0.01	0.05	0.3522	
**s.e.d.**		0.04	0.01	0.00	0.01	0.0459	
**significantly**	fallow	a	a	a	a	a	
**different**	arable	ab	a	b	a	b	
**means**	grass	b	a	b	a	b	
**comammox**	**mRNA ‰**	***N. inopinata***	**N.nitrificans**	**N. nitrosa**	**comammox**		
**ANOVA**	$F_{2,6}$	0.84	6.05	3.91	5.49		
	P	0.476	0.036	0.082	0.044		
	fallow	0.06	0.55	0.05	0.66		
**means**	arable	0.06	0.41	0.06	0.53		
	grass	0.04	0.09	0.03	0.16		
**s.e.d.**		0.01	0.14	0.01	0.16		
**significantly**	fallow	a	b	a	b		
**different**	arable	a	ab	a	ab		
**means**	grass	a	a	a	a		

Supplementary Table 4. ANOVA results and mean proportion of mRNA reads in groups as ‰ total prokaryotic sequences. Different letters denote significantly different means ($\alpha = 0.5$) according to Tukey's post-hoc method.

***amoA*-AOA**	**DNA g^{-1}**	**NS**	**NP**	**NT**	**NC**	***amoA*-AOA**
**ANOVA**	$F_{2,6}$	28.35	17.46	4.01	8.85	32.11
	P	<.001	0.003	0.111	0.023	<.001
**means**	fallow	150.4	30.41	5.163	4.8	190.8
	arable	1222	162.2	19.362	40.83	1444.4
	grass	443.9	40.71	4.887	45.3	534.8
**s.e.d.**		164	24.8	5.78	17.6	173.1
**significantly different means**	fallow	a	a	a	a	a
	arable	c	b	a	b	c
	grass	b	a	a	b	b
***amoA*-AOB**	**DNA g^{-1}**	***Nitrosospira***	***Nitrosomonas***	**comammox A**	**comammox B**	***amoA*-AOB**
**ANOVA**	$F_{2,6}$	44.97	23.63	6.63	1.25	32.38
	P	<.001	0.00	0.03	0.35	<.001
**means**	fallow	34.10	82.40	21.47	11.15	149.10
	arable	492.50	465.50	95.60	77.16	1128.30
	grass	90.80	305.90	22.92	45.80	471.10
**s.e.d.**		52.70	56.00	23.28	39.00	147.10
**significantly different means**	fallow	a	a	a	a	a
	arable	b	c	b	a	c
	grass	a	b	a	a	b
***amoA*-AOA**	**mRNA g^{-1}**	**NS**	**NP**	**NT**	**NC**	***amoA*-AOA**
**ANOVA**	$F_{2,6}$	16.22	4.55	1.69	101.96	10.01
	P	0.00	0.08	0.26	<.001	0.01
**means**	fallow	2222.00	358.00	102.00	77.70	2759.00
	arable	81652.00	4700.00	659.90	700.00	87711.00
	grass	16297.00	1155.00	840.00	0.00	18292.00
**s.e.d.**		14882.70	808.00	418.00	53.70	15535.30
**significantly different means**	fallow	a	a	a	a	a
	arable	b	a	a	b	b
	grass	a	a	a	a	ab
amoA-AOB	**mRNA g^{-1}**	***Nitrosospira***	***Nitrosomonas***	**comammox A**	**comammox B**	***amoA*-AOB**
**ANOVA**	$F_{2,6}$	22.67	16.51	11.73	9.28	16.07
	P	<.001	0.00	0.01	0.02	0.00
**means**	fallow	347.00	1417.00	574.00	168.70	2506.00
	arable	6594.00	44857.00	23345.00	438.70	75234.00
	grass	2112.00	10436.00	5246.00	875.50	18669.00
**s.e.d.**		956.60	7978.50	4967.00	165.60	13471.80
**significantly different means**	fallow	a	a	a	a	a
	arable	b	b	b	a	b
	grass	a	a	a	a	a

Supplementary Table 5. ANOVA results and mean DNA and mRNA reads assigned to *amoA* belonging to groups of AOA or AOB (including comammox) expressed as reads g^{-1} dw soil, totals for each guild in right-hand column. Different letters denote significantly different means (α = 0.5) according to Tukey's post-hoc method. *Nitrososphaera* and *Ca.* Nitrososcosmicus were not discriminated from the short reads and are grouped under NS.

***amoA*-AOA**	**DNA ‰**	**NS**	**NP**	**NT**	**NC**	***amoA*-AOA**
**ANOVA**	$F_{2,6}$	18.54	13.49	0.7	1.11	22.51
	P	0.003	0.006	0.55	0.4	0.002
	fallow	0.001761	0.000356	6.05E-05	5.61E-05	0.002233
**means**	arable	0.003347	0.000444	5.3E-05	0.000112	0.003956
	grass	0.0006	0.000055	6.61E-06	6.12E-05	0.000723
**s.e.d.**		0.000537	0.000108	2.88E-05	4.28E-05	0.000586
**significantly different means**	fallow	b	b	a	a	b
	arable	b	b	a	a	b
	grass	a	a	a	a	a
***amoA*-AOB**	**DNA ‰**	***Nitrosospira***	***Nitrosomonas***	**comammox A**	**comammox B**	***amoA*-AOB**
**ANOVA**	$F_{2,6}$	16.11	10.2	0.19	4.58	16.76
	P	0.004	0.012	0.828	0.122	0.004
	fallow	0.02601	0.004192	0.001195	0.00091	0.03231
**means**	arable	0.22364	0.012873	0.001807	0.001917	0.24024
	grass	0.02203	0.001561	0.001136	0	0.02473
**s.e.d.**		0.0406	0.00262	0.001193	0.000405	0.0422
**significantly different means**	fallow	a	a	a	ab	a
	arable	b	b	a	b	b
	grass	a	a	a	a	a
***amoA*-AOA**	**mRNA ‰**	**NS**	**NP**	**NT**	**NC**	***amoA*-AOA**
**ANOVA**	$F_{2,6}$	26.65	11.09	19.14	1.43	21.28
	P	0.001	0.01	0.005	0.312	0.002
	fallow	0.0004	0.000966	0.000252	0.000131	0.001749
**means**	arable	0.001394	0.001317	0.000271	0.000211	0.003193
	grass	0.000109	0.000368	2.76E-05	6.19E-05	0.000567
**s.e.d.**		0.000185	0.000238	6.98E-05	8.86E-05	0.000481
**significantly different means**	fallow	b	b	b	a	b
	arable	c	b	b	a	b
	grass	a	a	a	a	a
***amoA*-AOB**	**mRNA ‰**	***Nitrosospira***	***Nitrosomonas***	**comammox A**	**comammox B**	***amoA*-AOB**
**ANOVA**	$F_{2,6}$	24.96	18.31	12.87	0.32	18.09
	P	0.001	0.003	0.007	0.738	0.003
	fallow	0.004067	0.01662	0.00673	0.001979	0.0294
**means**	arable	0.018659	0.12694	0.06606	0.001241	0.2129
	grass	0.00254	0.01255	0.00631	0.001053	0.0251
**s.e.d.**		0.00252	0.02145	0.01355	0.001226	0.0359
**significantly different means**	fallow	a	a	a	a	a
	arable	b	b	b	a	b
	grass	a	a	a	a	a

Supplementary Table 6. Mean proportion of DNA and mRNA reads assigned to *amoA* belonging to groups as ‰ total prokaryotic sequences (guild totals on right) with ANOVA results. Different letters denote significantly different means ($\alpha = 0.5$) according to Tukey's post-hoc method. *Nitrososphaera* and *Ca.* Nitrososcosmicus were not discriminated from the short reads and are grouped under NS.
